# Hip Fracture Patients in Geriatric Rehabilitation Show Poor Nutritional Status, Dietary Intake and Muscle Health

**DOI:** 10.3390/nu12092528

**Published:** 2020-08-20

**Authors:** Inge Groenendijk, Charlotte S. Kramer, Laura M. den Boeft, Hans S.M. Hobbelen, Gert-Jan van der Putten, Lisette C.P.G.M. de Groot

**Affiliations:** 1 Division of Human Nutrition and Health, Wageningen University & Research, P.O. Box 17, 6700 AA Wageningen, The Netherlands; charlotte.kramer@wur.nl (C.S.K.); lisette.degroot@wur.nl (L.C.P.G.M.d.G.); 2 Amaris Zorggroep, 1251 CM Laren, The Netherlands; laura.denboeft@icloud.com; 3 Department of General Practice and Elderly Care Medicine, University of Groningen, University Medical Center Groningen, 9713 GZ Groningen, The Netherlands; j.s.m.hobbelen@pl.hanze.nl; 4 Research Group Healthy Ageing, Allied Health Care and Nursing, Hanze University of Applied Sciences, 9747 AS Groningen, The Netherlands; 5 Department of Oral Function and Prosthetic Dentistry, Radboud Medical Centre, 6525 GA Nijmegen, The Netherlands; gjvdputten@hetnet.nl; 6 Orpea, Dagelijks Leven, 7327 AA Apeldoorn, The Netherlands

**Keywords:** hip fracture, geriatric rehabilitation, nutritional status, dietary intake, muscle, handgrip strength, protein, energy

## Abstract

The aim of this study was to gain insight into the nutritional status, dietary intake and muscle health of older Dutch hip fracture patients to prevent recurrent fractures and to underpin rehabilitation programs. This cross-sectional study enrolled 40 hip fracture patients (mean ± SD age 82 ± 8.0 years) from geriatric rehabilitation wards of two nursing homes in the Netherlands. Assessments included nutritional status (Mini Nutritional Assessment), dietary intake on three non-consecutive days which were compared with Dietary Reference Intake values, and handgrip strength. Muscle mass was measured using Bioelectrical Impedance Analysis and ultrasound scans of the rectus femoris. Malnutrition or risk of malnutrition was present in 73% of participants. Mean energy, protein, fibre and polyunsaturated fat intakes were significantly below the recommendations, while saturated fat was significantly above the UL. Protein intake was <0.8 in 46% and <1.2 g/(kg·day) in 92%. Regarding micronutrients, mean intakes of calcium, vitamin D, potassium, magnesium and selenium were significantly below the recommendations. The prevalence of low muscle mass, low handgrip strength and sarcopenia were 35, 27 and 10%, respectively. In conclusion, a poor nutritional status, dietary intake and muscle health are common in older hip fracture patients in geriatric rehabilitation wards.

## 1. Introduction

Hip fractures (i.e., proximal femur fractures) are common injuries seriously affecting the health status and quality of life of older patients [1]. Within the following year, 22 percent of older hip fracture patients die and only 40 to 60 percent of the survivors regain their pre-fracture functional level [2,3]. Furthermore, the risk of reoccurring fractures persists for at least 10 years following the initial fracture [4], meaning that not only the initial fracture but also subsequent fractures should be an important focus for prevention.

Three major risk factors for getting a hip fracture are sarcopenia [5], osteoporosis [6] and malnutrition [7]. Sarcopenia, defined by low levels of muscle strength, muscle quantity/quality and physical performance [5], is common in older hip fracture patients with higher prevalence rates compared to older adults without a hip fracture [8,9]. Sarcopenia increases the risk of falls and fractures and has been associated with poorer functional recovery [10,11], increased probability of long-term care placement, and mortality [11]. While sarcopenia might already have been present prior to the fracture, an acute period of disuse of muscles during hospitalization is likely to induce further and rapid decline of muscle mass, strength and function [12,13,14].

Osteoporosis is a chronic disease characterized by low bone mass and deterioration of bone microarchitecture [15]. It increases the risk of falls and fractures, which in turn leads to an increase in morbidity and mortality, loss of independence, and a decreased quality of life [6]. After a hip fracture, an increased loss of bone mineral density (BMD) can be observed, which can continue for at least 1 year [16,17,18].

Older hip fracture patients are often malnourished or at risk of malnutrition [19,20,21]. Malnutrition can be caused by multiple factors including a reduced dietary intake (due to a lack of appetite, inability to eat or oral health problems), malabsorption, increased nutrient losses or altered metabolic requirements [22]. Malnutrition increases the risk of post-fracture complications; it is associated with delirium, an increase in mortality and comorbidities, a decline in mobility, and it prolongs rehabilitation [21,23,24,25].

Energy and protein requirements are increased in hip fracture patients; they often have a lower calorie intake plus an increased energy requirement due to an inflammatory state [21]. For protein, there is no official recommendation specifically for patients recovering from a hip fracture, but an intake of 1.2–1.5 g/(kg·day) for older people with a severe illness or injury can be derived from consensus papers [26,27]. With respect to micronutrients, a sufficient intake of vitamin D and calcium are essential for musculoskeletal health [28,29,30]. Furthermore, there might be a role of vitamin K in reducing fracture risk, but there is no clear evidence [31].

Previous studies have shown that indeed energy, protein, vitamin D and calcium intakes can be low after a hip fracture [32,33,34,35]. However, these studies involve hospitalized patients and still little is known about inpatient geriatric rehabilitation. Furthermore, intakes may also be country-specific, therefore, results cannot always be translated. In addition, a broad overview of the characteristics of this population, from nutritional status to dietary intake to muscle health, is missing.

In order to prevent recurrent fractures and to create the most optimal rehabilitation program, more knowledge is needed on the characteristics of older patients recovering from a hip fracture. The aim of this study was to gain insight into the nutritional status, dietary intake and muscle health of older hip fracture patients in geriatric rehabilitation wards in the Netherlands.

## 2. Materials and Methods

### 2.1. Study Population

A cross-sectional study was conducted at geriatric rehabilitation (GR) wards of two Dutch nursing homes. The study population consisted of 40 older adults (≥60 years) admitted to the GR ward after hospital treatment (conservative or surgical) for a hip fracture. A triage has taken place in the hospital to determine if the person was suitable for rehabilitation (i.e., trainable). No exclusion criteria were applied, and as a result, all patients admitted to these wards were invited to participate within the first three days after admission. Measurements were conducted during the first week of admission. The study was conducted in accordance with the Declaration of Helsinki, and the Medical Ethics Committee of Wageningen University gave ethical exemption for this study. All participants gave their informed consent for inclusion before they participated in the study.

### 2.2. Nutritional Status and Dietary Intake

Nutritional status was assessed using the 18-item Mini Nutritional Assessment (MNA). The MNA categorizes patients as either having a good nutritional status (24–30 points), being at risk of malnutrition (17–23.5 points) or as malnourished (<17 points) [36].

Dietary intake was recorded within the first week after admission on three non-consecutive days, including two weekdays and one weekend day. Trained researchers filled out food records through a combination of observations and weighing. The assortment available at the nursing homes for breakfast and lunch were weighed at the beginning of the study. Soup, desserts and each component of hot meals (and leftovers) were weighed using a kitchen scale (Kern EMB 2200-0, Kern & Sohn GmbH, Balingen, Germany) before and after the participant consumed them. Data were processed with Compl-eat™ (Department of Human Nutrition and Health, Wageningen University, Wageningen, the Netherlands), which was linked to the Dutch food composition database NEVO-online version 2016 [37].

Dietary intakes were compared with Dietary Reference Intake (DRI) values [38], the Estimated Average Requirements (EAR) and the Recommended Dietary Allowances (RDA). When these were not available, the Adequate Intake (AI) was used. Saturated fat, polyunsaturated fat and sodium did not have a recommended level of intake but only a Tolerable Upper Intake Level (UL). Macro- and micronutrient norms were based on guidelines of the Dutch Health Council [39,40,41]. The Dutch Health council only issued an advice for vitamin K1 and not total vitamin K. Therefore, the AI for total vitamin K intake were based on the recommendation of the American Institute of Medicine [42]. Energy intake (g/(kg·day)) and protein intake (g/(kg·day)) were compared with recommendations based on expert groups (European Society for Clinical Nutrition and Metabolism (ESPEN) and the PROT-AGE Study Group) [27,43].

Protein intake per main meal was calculated to investigate protein distribution over the day. For older adults it is recommended to consume 25 to 30 g protein per main meal [27]. Since one nursing home served warm meals at dinner and the other nursing home at lunchtime, data were categorized as warm/cold meals instead of lunch/dinner moment.

### 2.3. Muscle Health

Sarcopenia was defined as the presence of both low Appendicular Skeletal Muscle Mass (ASMM) and low handgrip strength [5]. ASMM was measured using Bioelectrical Impedance Analysis (BIA) with a 50 kHz single frequency impedance meter (BodyExplorer PAD, Juwell Medical GmbH, Rheine, Germany) on the non-fractured side. The protocol by Kyle was used: in a fasted state (in the morning with patient still in bed), in a supine position with electrodes on the hand and foot, having rested with no exercise for >8 h and the bladder voided. Participants with pacemakers or implanted defibrillators were excluded from the BIA measurement because of possible electromagnetic interference [44]. For the calculation of ASMM, the equation developed by Sergi was used [45]. As recommended by the European Working Group on Sarcopenia in Older People 2 (EWGSOP2), low ASMM was defined as <20 kg for men and <15 kg for women [5]. Subsequently, ASMM was divided by height squared (m^2^) to adjust for body size. Corresponding cut-off points were <7.0 kg/m^2^ for men and <5.5 kg/m^2^ for women [5].

Handgrip strength was measured with a JAMAR hydraulic handheld dynamometer (model 5030J1, Sammons Preston, Bolingbrook, IL, USA) in seated position with the elbows flexed at 90 degrees. During three trials on each hand, participants were verbally encouraged to produce their maximum grip strength. The highest value was used for analysis [46]. EWGSOP2 cut-off points were used to indicate poor muscle strength: <27 kg for men and <16 kg for women [5].

Ultrasound scans of the rectus femoris were conducted using the HS-2200 (Honda, Toyohashi, Aichi, Japan) with the 7.5 MHz linear transducer probe. Patients were in supine position with extended knees. The middle between the anterior inferior iliac spine and the midpoint of the proximal border of the patella and the one-third point seen from the patella were marked using a measuring tape. The ultrasound was performed by positioning the probe at the one-third point with minimal pressure in a transversal position. The thickness of the rectus femoris was measured by hand using the measurement function of the HS-2200. The thickness was measured from the transition of fascia to muscle, to the transition of muscle to fascia at the muscle’s thickest point. Cut-off points for low muscle mass were defined as 2SD below the gender-specific mean of a younger reference group: RF <19.9 and <15.9 mm in men and women, respectively [47].

### 2.4. Demographics, Medical and Physical Details

Demographic and medical data (comorbidities, number of medication use, estimated Glomerular Filtration Rate (eGFR), details about the fracture) were obtained from medical records. In addition, the Charlson Comobidity Index (CCI, 0–37 points) was used to determine the number and severity of comorbidities [48]. The higher the score, the more comorbidities the participant suffers from. The presence of a delirium was assessed according to Delirium Observation Screening (DOS, 0–13 points). A DOS score of ≥3 indicates delirium [49].

The Evaluative Frailty Index for Physical activity (EFIP) was used to determine pre-fracture frailty status [50]. The EFIP is a 50-item questionnaire that includes the physical, psychosocial and social domain and general health status. The score is calculated by dividing the total score by 50. A cut-off score of 0.2 was used to indicate frailty [51].

The Barthel Index (BI, 0–20 points) was used to evaluate the level of independence in activities of daily living at the time of admission with higher scores indicating a higher level of independence [52]. Lastly, walking ability was measured using the Functional Ambulation Categories (FAC, 0–5 points) [53]. A score of 0 indicates no ambulation or non-functional walking and 5 indicates independent walking on all surfaces and able to climb stairs.

Body weight was measured to the nearest 0.1 kg using either a digital weighing scale (Seca 876, Seca Ldt, Birmingham, UK) or wheelchair scale (Seca 677, Seca Ldt, Birmingham, UK) depending on the participants ability to stand. Height was measured to the nearest 1 cm in standing position with a wall-mounted stadiometer (Seca, Seca Ldt, Birmingham, UK). In case the participant was not able to stand straight, total body height was calculated from knee height using the LASA formula, which is developed based on data from a Dutch cohort of older people [54].

### 2.5. Statistical Analysis

Continuous data are expressed as mean ± standard deviation (SD) or as median with interquartile range (IQR) in case of a non-normal distribution. Categorial variables are presented as number of participants with percentage. All data were checked for normality. Outliers (±3 SD from mean) in primary dependent variables were retained in final analyses when results including and excluding the outlier were similar.

To test whether the mean of a single dietary intake variable differed from the recommendation, a one-sample *t*-test was used. Micronutrient intakes which could not be compared with the EAR were classified as inadequate when statistically significant and <67% of the RDA or AI. In case of a non-normal distribution, Wilcoxon signed-rank test was used. Comparisons between the two study sites for patient characteristics and dietary intake were made using an independent sample *t*-test or the Mann–Whitney test.

All statistical analyses were carried out using IBM SPSS statistics (version 25.0; SPSS, Chicago, IL, USA). A two-sided *p*-value of 0.05 was used for statistical significance.

## 3. Results

### 3.1. Study Population

Between October 2017 and April 2018, 44 hip fracture patients were admitted to the GR wards of which 40 patients (91%) were included. Reasons for not participating were not interested in participation (*n* = 3) and readmission to the hospital within two days (*n* = 1). Participant characteristics are described in Table 1. Before the hip fracture, 27 participants lived independently, 11 participants received home care and 2 participants lived with their family. The majority of participants suffered a fragility hip fracture (i.e., caused by a fall from standing height or less) and three participants suffered from a high impact fracture (i.e., traffic accident). Median length of stay in the hospital was five days. After admission to the GR wards, all assessments were completed in a median of four days (varying from 2 to 10 days). There were no statistically significant between-group differences in participant characteristics with regards to the two study sites.

### 3.2. Nutritional Status and Dietary Intake

Three participants (8%) were categorized as being malnourished and a further 26 participants (65%) as being at risk of malnutrition. Half of the participants had contact with a dietician at admission.

Three-day records were completed for 31 participants and two days were completed for the remaining nine. The latter was due to discharge home (*n* = 3), illness (*n* = 3), readmission to the hospital (*n* = 2) and closing of the GR ward because of gastroenteritis virus (*n* = 1). One participant had to be excluded because of outliers in the dietary intake variables.

**Figure 1:**
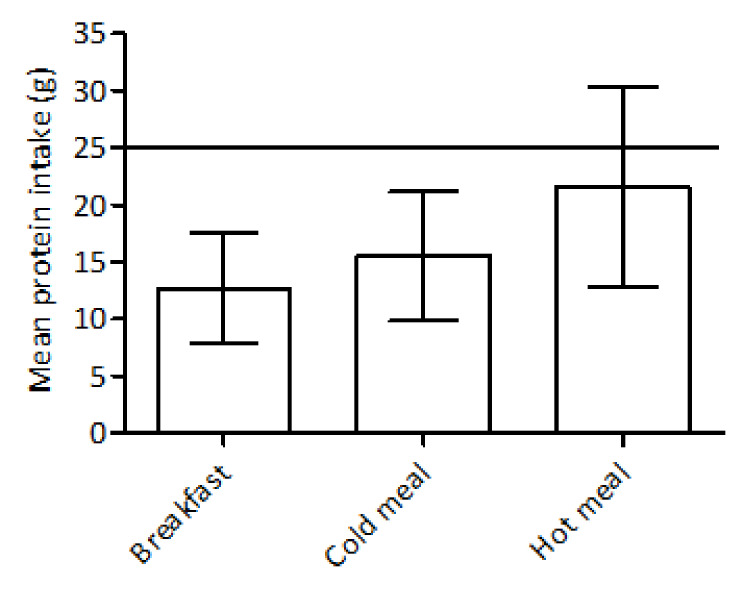
Protein intake of 39 older hip fracture patients per main meal. Values are means ± SD. Horizontal line represents the recommend lower limit of protein intake per main meal.

Energy, protein, fibre and polyunsaturated fat intakes were below the recommendations, while saturated fat was significantly above the UL (Table 2). The mean daily protein was 0.82 ± 0.28 g/(kg·day)) and ranged from 0.25 to 1.55 g/(kg·day). The percentage of participants with an insufficient intake of <0.8, <1.0 and <1.2 g/(kg·day) amounted to 46, 74 and 92%, respectively. Mean protein intake (g) was below the recommended 25 to 30 g protein for each main meal (Figure 1). With respect to the micronutrient intakes, mean/median intakes of calcium, vitamin D, potassium, magnesium and selenium were significantly below the recommendations (Table 3).

### 3.3. Muscle Health

BIA was performed in 37 participants; data were missing for three participants because of the presence of a pacemaker or implanted defibrillator. Low ASMM was present in 13 participants (35%). Handgrip strength measurements were completed for 37 participants, it was low in 10 participants (27%). Of the 35 participants with complete data, 10% had sarcopenia.

Mean muscle thickness of the rectus femoris measured with ultrasound was 10.5 ± 2.2 mm at the fractured site (*n* = 32) and 10.8 ± 2.0 mm and the non-fractured site (*n* = 34). For all participants with measurements, this was classified as being low. One outlier was removed from the ultrasound results of both the fractured as unfractured site.

## 4. Discussion

This study showed that along with a high prevalence of malnutrition (risk), nutrient intake was poor in hip fracture patients. Patients had low protein, energy, fibre and polyunsaturated fat intakes and a high saturated fat intake. In addition, intakes of several micronutrients were well below the recommendations. Approximately one third had a low muscle mass and a quarter showed low muscle strength.

The majority of the participants, 73%, were classified as either malnourished or at risk of malnutrition. In other studies that used the MNA to measure nutritional status in hip fracture patients, prevalence varied from 30 to 86% for malnourishment and risk of malnutrition together [20,23,32,33,34,55,56,57]. Differences in prevalence may be explained by the inclusion of patients with dementia and delirium as we did in our study, because these patients have an increased risk of malnutrition [58,59,60]. Since most nutrient intakes were low relative to the recommendations, the prevalence of (risk of) malnutrition may even further increase if patients do not receive nutritional support.

One of the striking findings was that the percentage of participants with an insufficient protein intake of <0.8, <1.0 and <1.2 g/(kg·day) amounted to 46, 74 and 92%, respectively. Such low protein intake can further induce a decline of muscle and bone mass, a higher hip fracture risk and a poor nutritional status [26,61]. Other studies in hip fracture patients found mean protein intakes ranging from 43 to 57 g [33,34,35,62], which are comparable to our findings (55 g). In these studies energy intakes were also low (mean intakes ranged from 1025 to 1304 kcal versus 1319 kcal in the current study) [33,34,35,62]. Since the intake of protein and energy were low (overall low intake of food), the intakes of many other nutrients were low as well. Therefore, nutritional support should primarily focus on increasing nutrient-dense foods. People with severe kidney problems should avoid a high protein intake, because this can be harmful [63]. In the current study, kidney failure was not present and only one participant had severe loss of kidney function (eGFR < 30 mL/min). Therefore, increasing the protein intake in this population seems (in general) feasible.

Micronutrients of concern include calcium, vitamin D, potassium, magnesium and selenium. Note that for these micronutrients, with the exception of vitamin D, intakes could only be compared with an adequate intake. However, these nutrients were <67% of this norm. Especially a sufficient intake of calcium and vitamin D are important in this population, because these nutrients are essential for musculoskeletal health [28,29,30]. A sufficient calcium intake may already be reached by increasing the protein intake through the consumption of more dairy products, vegetables and/or nuts. Median vitamin D intake (1.9 µg) was far below the EAR (10 µg) and RDA (20 µg). However, supplementation was not taken into account as this information was not available. The Dutch Health Council advices people aged ≥70 years to take a daily vitamin D supplement of 20 µg [64] and staff of the two nursing homes stated that indeed vitamin D supplements are given to hip fracture patients. If this is truly the case, vitamin D intake would be sufficient.

We also showed that low muscle mass was present in 35% of the participants according to BIA, while this was 100% according to the muscle thickness of the rectus femoris measured with ultrasound. One possible explanation for this discrepancy is that BIA is known to overestimate muscle mass (giving lower prevalence rates of low muscle mass) [65,66]. Where BIA estimates muscle mass, ultrasound measures only the size of one muscle. Ultrasound shows good validity for measuring muscle size in older adults, but how this relates to overall muscle mass is unclear [67]. Considering that sarcopenia affects various muscles at different rates, sarcopenia seems more prevalent in the lower limb muscles [68]; multiple studies suggest that sarcopenia prevalence is higher when measuring thigh muscles than when measuring multiple body sites [69] and that the rectus femoris might decline specifically early [70]. Even though there are methodological considerations for each method, we can nevertheless conclude with relative certainty that the prevalence of low muscle mass was substantial (with a true prevalence being at least 35%).

Furthermore, handgrip strength was low in 27% of the participants, which is comparable to previous studies where it varied from 14 to 27% [10,11,32,71]. Combining the low muscle mass measured with BIA and the low handgrip strength, 10% of the participants had sarcopenia. Although this percentage is not as high when considering muscle mass and strength separately, we should still be aware of the fact that patients are in a post-fracture catabolic state and may develop sarcopenia in a later stage. Besides pre-existing malnutrition, muscle mass may further decline by the inflammatory response after a hip fracture. The post-fracture catabolic state may continue for up to 3 months [72]. Prevalence of sarcopenia found in previous studies in hip fracture patients were quite diverse, ranging from 17 to 87% [10,11,32,71,73,74,75,76]. To minimize adverse outcomes like loss of muscle mass and mobility impairment, nutritional interventions in combination with resistance exercise may offer a solution.

In addition to this, the large variety in characteristics such as muscle mass and strength, cognitive and physical status and dietary intake in older hip fracture patients point to a compelling need for individually tailored interventions. For instance, 20% of participants experienced a delirium, 13% had dementia, 43% had a cardiac comorbidity, 20% had diabetes and 20% were not allowed to fully bear weight on the fractured leg. Therefore, some patients will need more supervision, modified exercises or different nutritional support. We advise that every new hip fracture patient admitted to a geriatric rehabilitation ward is guided by an interprofessional team of a physician, physiotherapist, nurse and dietician. Furthermore, monitoring dietary intake in geriatric rehabilitation is important. Particular attention should be paid to the amount of protein intake and nursing staff should be made aware of the importance of sufficient protein intake in patients in a geriatric rehabilitation ward. Oral health should be taken into account as well, since poor oral health can lead to a reduced nutrient intake and malnutrition [77].

A limitation of this study is that there were missing data for some participants, because certain assessment tools, like BIA, were unsuitable for the participants. It remains a challenge to perform a variety of measurements on frail older adults. Sarcopenia cut-off points for low strength and low muscle quantity were according to the latest consensus EWGSOP2 [5], but we did not include a test to identify low performance levels (many patients are not able to perform such tests within the first couple of days after the hip fracture due to pain). Therefore, diagnosis of sarcopenia was not completely according to the consensus and we should be careful with comparing our sarcopenia prevalence with that in other studies. Lastly, this study was only conducted in two Dutch nursing homes, raising the question if this is representative of other centres in the Netherlands. The included population covered the entire target population of the two studied locations as 91% of hip fracture patients were willing to participate. Moreover, referral to geriatric rehabilitation wards is preceded by triage under the responsibility of an elderly care physician by using uniform criteria for all wards in the Netherlands [78]. This uniform selection, in addition to the high percentage of participation in the current study, suggests that the studied population is representative for hip fracture patients in geriatric rehabilitation wards in the Netherlands, despite the relatively small sample size.

In line with this, a strength of this study is that there were no exclusion criteria, which gives a better representation of the target population. Another strength is the accurate assessment of dietary intake by the combination of direct observations and weighing on three non-consecutive days. This method reduces the chance of measurement errors and recall bias compared to methods like 24-h recalls.

## 5. Conclusions

This study showed that hip fracture patients in geriatric rehabilitation have a poor nutritional status, dietary intake and muscle health. It is recommended to offer a postoperative personalized nutritional intervention with longitudinal follow-up to these patients with special attention to increase energy and protein intake. Such an intervention in combination with exercise may prevent recurrent fractures, reduce morbidity and optimise recovery after a hip fracture.

## Figures and Tables

**Table 1:** Characteristics of the hip fracture patients.

Characteristic	*n*	Value
Women, n (%)	40	29 (73)
Age, mean ± SD, year	40	81.6 ± 8.0
Weight, median (IQR), kg	40	68.0 (56.2–79.9)
Height, mean ± SD, cm	40	165 ± 9
BMI, median (IQR), kg/m^2^	40	24.8 (21.5–28.5)
No weight bearing, n (%)	40	8 (20)
Surgical method	40	
Prosthetic replacement, n (%)		14 (35)
Internal fixation, n (%)		23 (58)
None, conservative, n (%)		3 (8)
Barthel index, mean ± SD, points	40	10.1 ± 3.9
FAC, points	40	
0, n (%)		18 (45)
1, n (%)		0 (0)
2, n (%)		2 (5)
3, n (%)		10 (25)
4, n (%)		10 (25)
5, n (%)		0 (0)
Delirium (DOS > 3), n (%)	40	8 (20)
Frail (EFIP > 0.2), n (%)	36	12 (33)
Kidney function (eGFR)	37	
Kidney failure (<15 mL/min), n (%)		0 (0)
Severe loss (<30 mL/min), n (%)		1 (3)
Moderate loss (30–60 mL/min), n (%)		8 (22)
Mild loss (60–90 mL/min), n (%)		19 (51)
Normal (>90 mL/min), n (%)		9 (24)
CCI, median (IQR), points	40	1 (0–2)
Comorbidity	40	
Diabetes Mellitus, n (%)		8 (20)
Cardiac, n (%)		17 (43)
Pulmonary, n (%)		8 (20)
Dementia, n (%)		5 (13)
Previous fracture due to fall	40	12 (33)
MNA	40	
Malnourished, n (%)		3 (8)
Risk of malnutrition, n (%)		26 (65)
Good nutritional status, n (%)		11 (28)
ASMM, median (IQR), kg	37	16.5 (15.2–18.9)
Low ASMM, n (%)		13 (35)
ASMM/height^2^, mean (SD), kg/m^2^	37	6.4 ± 1.0
Low ASMM/height^2^, n (%)		13 (35)
Handgrip strength, mean ± SD, kg	37	22.5 ± 9.3
Low handgrip strength, n (%)		10 (27)
Sarcopenia, n (%)	35	4 (10)

Values are frequency (percentage), mean ± SD, or median (IQR). ASMM = Appendicular Skeletal Muscle Mass; BMI = Body Mass Index; CCI = Charlson Comorbidity Index; DOS = Delirium Observation Screening; EFIP = Evaluative Frailty Index for Physical activity; eGFR = estimated Glomerular Infiltration Rate; FAC = Functional Ambulation Categories; IQR = interquartile range; MNA = Mini Nutritional Assessment; SD = standard deviation.

**Table 2:** Daily mean macronutrient intake of 39 older hip fracture patients compared to the Dietary Reference Intakes from European Society for Clinical Nutrition and Metabolism (ESPEN) [43], the PROT-AGE Study Group [27] and the Health Council of the Netherlands (RDA, AI, UL) [39,40].

Macronutrient	Intake	DRI		*p* Value ^2^
Energy, kcal	1319 ± 285	-		
Energy, kcal/kg bw	19.7 ± 6.1	30	ESPEN	<0.001
Protein, g	54.9 ± 14.3	60/51 ^1^	RDA	0.033/0.095
Protein, g/kg bw	0.82 ± 0.28	1.0–1.2	ESPEN/PROT-AGE	<0.001
Protein, en%	17.5 ± 3.6	11	RDA	<0.001
Carbohydrates, g	128.1 ± 34.6	-		
Carbohydrates, en%	40.7 ± 7.3	40	RDA	0.56
Fibre, g	12.4 ± 3.9	-		
Fibre, g/MJ	2.3 ± 0.5	3.4	AI	<0.001
Fat, g	60.9 ± 15.0	-		
Fat, en%	41.4 ± 4.7	20–40	AI	<0.001–0.063
Saturated fat, g	29.3 ± 8.3	-		
Saturated fat, en%	19.9 ± 3.6	10	UL	<0.001
Monounsaturated fat, g	15.8 ± 4.6	-		
Polyunsaturated fat, g	8.4 ± 2.9	-		
Polyunsaturated fat, en%	5.8 ± 1.8	12	UL	<0.001

Data are presented as mean ± SD. AI = Adequate Intake; bw = body weight; DRI = Dietary Reference Intakes; EAR = Estimated Average Requirement; en% = energy percentage; UL = Tolerable Upper Intake Level; RDA = Recommended Dietary Allowance; - = no value established. ^1^ Men and women, respectively. ^2^ *p* value by one-sample *t*-test to analyse differences between mean intake and DRI.

**Table 3:** Daily micronutrient intake of 39 older hip fracture patients compared to the Dietary Reference Intakes from the Health Council of the Netherlands [41] and American Institute of Medicine (vitamin K) [42].

Micronutrient	Intake	DRI		Intake in % of DRI	*p* Value ^2^
Calcium, mg	718 ± 287	1200	AI	60	<0.001
Vitamin D, µg	1.8 (1.2–2.4)	20	RDA	9	<0.001
		10	EAR	18	<0.001
Vitamin K, µg	109 (51–203)	120/90 ^1^	AI	91/121 ^1^	0.62/0.027
Phosphorus, mg	972 ± 271	550	AI	177	<0.001
Iron, mg	6.2 ± 1.9	11/16 ^1^	RDA	56/39 ^1^	<0.001
		6	EAR	103	0.49
Natrium, mg	1657 ± 455	2400	UL	69	<0.001
Potassium, mg	1875 ± 465	3500	AI	54	<0.001
Magnesium, mg	186 ± 46	350/300 ^1^	AI	53/62 ^1^	<0.001
Zinc, mg	7.5 ± 2.3	9/7 ^1^	RDA	83/107 ^1^	<0.001/0.19
		6.4/5.7 ^1^	EAR	117/132 ^1^	0.006/<0.001
Selenium, µg	27.3 (21.5–35.0)	70	AI	39	<0.001
Copper, mg	0.66 ± 0.16	0.9	RDA	73	<0.001
		0.7	EAR	94	0.11
Iodine, µg	118 ± 43	150	AI	79	<0.001
Vitamin B12, µg	3.0 ± 1.1	2.8	RDA	107	0.27
		2.0	EAR	150	<0.001
Vitamin C, mg	56.0 (35.3–74.7)	75	RDA	75	0.002
		60	EAR	93	0.76

Data are presented as mean ± SD or median (IQR). AI = Adequate Intake; DRI = Dietary Reference Intakes; EAR = Estimated Average Requirement; UL = Tolerable Upper Intake Level; RDA = Recommended Dietary Allowance. ^1^ Men and women, respectively. ^2^ *p* value by one-sample *t*-test or Wilcoxon signed-rank test to analyse differences between mean intake and DRI.
